# Prescribed Drugs and Self-Directed Violence: A Descriptive Study in the Spanish Pharmacovigilance Database

**DOI:** 10.3390/ph16050772

**Published:** 2023-05-22

**Authors:** Ana Avedillo-Salas, Javier Pueyo-Val, Ana Fanlo-Villacampa, Cristina Navarro-Pemán, Francisco Javier Lanuza-Giménez, Ignatios Ioakeim-Skoufa, Jorge Vicente-Romero

**Affiliations:** 1 Department of Pharmacology, Physiology and Legal and Forensic Medicine, Faculty of Medicine, University of Zaragoza, ES-50009 Zaragoza, Spain; 2 Aragon Pharmacovigilance Center, ES-50017 Zaragoza, Spain; 3 WHO Collaborating Centre for Drug Statistics Methodology, Department of Drug Statistics, Division of Health Data and Digitalisation, Norwegian Institute of Public Health, NO-0213 Oslo, Norway; 4 EpiChron Research Group, Aragon Health Research Institute (IIS Aragón), Miguel Servet University Hospital, ES-50009 Zaragoza, Spain; 5 Research Network on Chronicity, Primary Care, and Health Promotion (RICAPPS), Institute of Health Carlos III (ISCIII), ES-28029 Madrid, Spain; 6 Drug Utilization Work Group, Spanish Society of Family and Community Medicine (semFYC), ES-08009 Barcelona, Spain

**Keywords:** adverse drug reactions, self-directed violence, pharmacovigilance, FEDRA^®^

## Abstract

Self-inflicted violence is a major and growing public health problem and its prediction and prevention is challenging for healthcare systems worldwide. Our aim was to identify prescribed drugs associated with self-directed violent behaviors in Spain. A descriptive, longitudinal and retrospective study of spontaneous reports of adverse drug reactions corresponding to self-directed violence was recorded in the Spanish Pharmacovigilance Database (FEDRA^®^) from 1984 to 31 March 2021. A total of 710 cases were reported in the study period. The mean age was 45.52 years (range 1–94). There were no gender differences except in children, where most reports were of male children. The main therapeutic groups that were involved included drugs for the nervous system (64.5%) and anti-infectives for systemic use (13.2%). The most commonly reported drugs were varenicline, fluoxetine, lorazepam, escitalopram, venlafaxine, veralipride, pregabalin, roflumilast and bupropion. There were reports of montelukast, hydroxychloroquine, isotretinoin, methylphenidate, infliximab, natalizumab, ribavirin and efavirenz, which were less known to be involved in self-directed violence. This study shows that self-directed violence is a rare adverse drug reaction, and can be related to the use of some medicines. It is important for healthcare professionals to consider this risk in their clinical praxis, implementing person-centred approaches. Further studies are needed, considering comorbidities and potential interactions.

## 1. Introduction

### 1.1. Background

The World Health Organization (WHO) defines violence as “the intentional use of physical force or power, threatened or actual, against oneself, another person, or against a group or community, that either results in or has a high likelihood of resulting in injury, death, psychological harm, maldevelopment or deprivation” [1,2]. There are three types of violence, depending on who commits the violent act: self-directed violence, interpersonal violence and collective violence (social, political and economic) [1].

The Centers for Disease Control (CDC) define self-directed violence as behavior that is self-directed and deliberately results in injury or the potential for injury to oneself [3]. This involves both self-injury and suicidal behavior. The main difference between non-suicidal self-injury and suicidal behaviors is the intention to end one’s own life.

**Figure 1:**
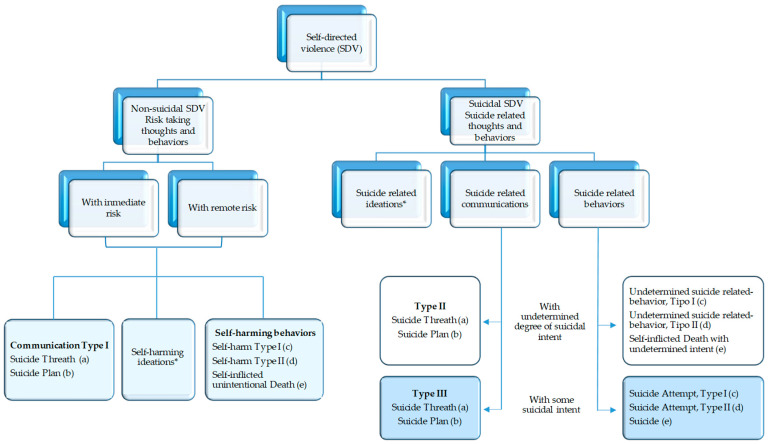
Self-directed violence classification. * Suicide related ideations can be casual, transient, passive or persistent independent of the degree of suicidal; (a) verbal or non-verbal, passive or active; (b) a proposed method of achieving a potentially self-injurious outcome; (c) without injuries; (d) with injuries; (e) with fatal outcome.

Self-directed violence includes suicidal ideations, communications and behaviors according to the Silverman et al. classification [4,5], in which the result of the behavior, the entity of the act, the degree of intentionality and the knowledge or awareness of the perpetrators are taken into account as results of this behavior [6], as can be seen in Figure 1.

Acts of self-injurious violence can be interrupted or suspended by the subject or by another person. The interruption can occur at any time during the act, such as after the initial thought or after the initiation of the behavior [7]

At the same time, self-directed violence can be physical, psychological or involve deprivation or neglect [1].

### 1.2. Self-Directed Violence: A Public Health Problem

Self-directed violence is a major public health problem worldwide and its prediction and prevention represent a challenge for everyone [8]. It is a leading cause of death and disability. Suicide, globally, causes more deaths than HIV, malaria, breast cancer, war or homicide. More than 700,000 people die by suicide every year globally: a rate of one suicide every 40 s. Suicides account of 1.4% of premature deaths worldwide [9]. Suicide was the fourth leading cause of death among 15–29 years old in 2019 [10]. In addition, it is estimated that 20 suicide attempts are made for every suicide.

In the European Union, 10.8 suicide deaths per 100,000 inhabitants were recorded, and mortality rates from intentional self-harm were 3.8 times higher among men than among women [11]. The lowest standardized mortality rates for suicide in 2017 were recorded in Cyprus (4.1 per 100,000 population), Greece and Malta (4.5 and 4.6 deaths per 100,000 population, respectively) [11].

In Spain, suicide is the leading cause of non-natural death (8.32 suicide deaths per 100,000 inhabitants), producing 2.7 times more deaths than traffic accidents and 13.6 times more than homicides [12]. Suicide attempts and self-harm have increased by 250% in Spain’s young population, and suicidal thoughts have also increased between 8% and 10%, according to the Mental Health and COVID-19 study conducted by the Confederation of Mental Health Spain. In 2020, 3941 suicides took place in Spain, of which 73.4% were men and 25.3% women. This represented an increase of 7.35% compared to 2019. The highest number of suicides occurred between 40 and 59 years of age (1608 suicides; 41% of the total). Moreover, with 300 deaths, suicide is, after tumors, the leading cause of death among Spanish youth (15–29 years) [13]. Consequently, the Spanish and world populations live with violence. Death represents the most extreme and infrequent manifestation of self-inflicted violence, but much more frequently its victims survive, leaving a wide range of bodily and functional alterations or causing persistent and serious psychological disorders. This leads to an increase in mortality and morbidity in society.

Reducing countries’ suicide rates has been a priority of the World Health Organization (WHO) and is an indicator in the United Nations Sustainable Development Goals (SDGs) (target 3.4), the WHO’s 13th General Programme of Work 2019–2023 (WHO GPW13) and the Mental Health Action Plan 2013–2022, which has been extended to 2030 [10].

Suicide screening (detection), timely registration and regular follow-up are key pillars of effective national suicide prevention strategies to identify specific suicide risk groups, address the needs of specific populations and understand the main risk factors for suicide [8].

### 1.3. Risks Factors

Self-directed violence is highly complex and multifaceted. A combination of situations (social, psychological, biological, economic, cultural and others) could lead someone to consider it.

Many risk factors have been extensively studied, such as individual risk factors (previous suicide attempts, serious or mental illness, economic or employment problems, social isolation, drug addiction, etc.), inadequate close social relationships with friends, partners and family members (child abuse, neglect in elder care, gender violence, etc.), community contexts (bullying and cyberbullying, limitations in accessibility to health care, sexual violence, certain beliefs of cultural and religious groups, such as altruistic suicide, etc.) and social stigmas attached to mental disorders and self-inflicted violence that make many people feel unable to seek help [2,8,14,15].

However, there is one risk factor for which there are few studies: the use of drugs [16,17,18], even though, in the Summary of Product Characteristics (SmPC) several of them list the possibility of developing self-inflicted violent behavior during their use as an adverse drug reaction. There are several studies that have analysed the relationship between certain drugs and pharmacological groups and self-directed violence [19,20,21,22,23,24,25], but there are few studies (none in Spain) that have analysed this relationship by encompassing all drugs marketed in a country [16,17,18,19,20,21,22,23,24,25,26]. An in-depth study of this risk factor, especially in an ageing population, is of high importance considering the continuously increasing prevalence of multimorbidity and polypharmacy and the potential drug–drug and drug–disease interactions.

### 1.4. Aim

In order to investigate the little-known role that drugs can pay in self-directed violence, the objective of this study was to analyze self-directed violence as an adverse drug reaction, at the national level, based on spontaneous reports of suspected adverse drug reactions from the Spanish Human Pharmacovigilance System Database (FEDRA^®^) from 1984 to 31 March 2021.

## 2. Results

In the study period, the Spanish Human Pharmacovigilance System received 353,165 spontaneous ADRs, of which 710 were spontaneous ADRs of self-inflicted violence (0.20%).

### 2.1. General Data on Reports

**Figure 2:**
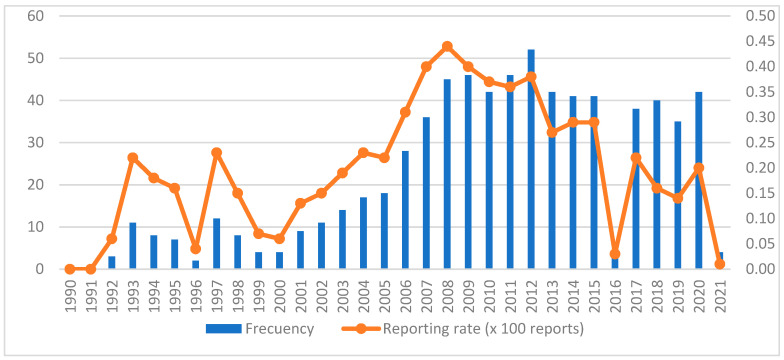
Temporal evolution of reports of self-inflicted violence.

The 710 reports were mostly received from the year 2007 onwards (*n* = 554; 78%), with the year 2012 standing out (*n* = 52; 7.3%). However, the year with the highest notification rate was 2008 (Figure 2).

The percentage of out-of-hospital and in-hospital reports was similar, at 36.7% (*n* = 273) and 37.4% (*n* = 278), respectively. The professionals who reported the most were physicians (*n* = 516; 69.4%) and pharmacists (*n* = 107; 14.4%).

**Figure 3:**
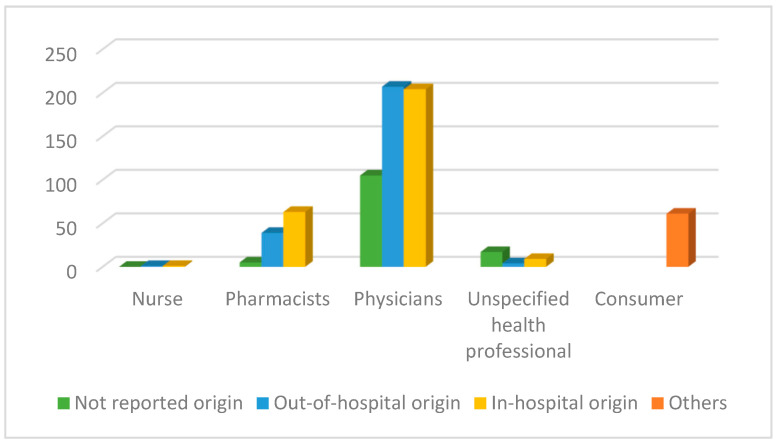
Number of reports according to primary sources.

Analyzing the origin of the notifier, in-hospital pharmacists notified 58.90% (*n* = 63) of the cases compared to 36.40% of out-of-hospital pharmacists, and 4.70% (*n* = 5) were not reported. Out-of-hospital physicians reported 40.10% (*n* = 207) of the cases, in-hospital physicians 39.5% (*n* = 204), and not reported 20.30% (*n* = 105), as shown in Figure 3.

### 2.2. Patient Data (Study Population)

**Figure 4:**
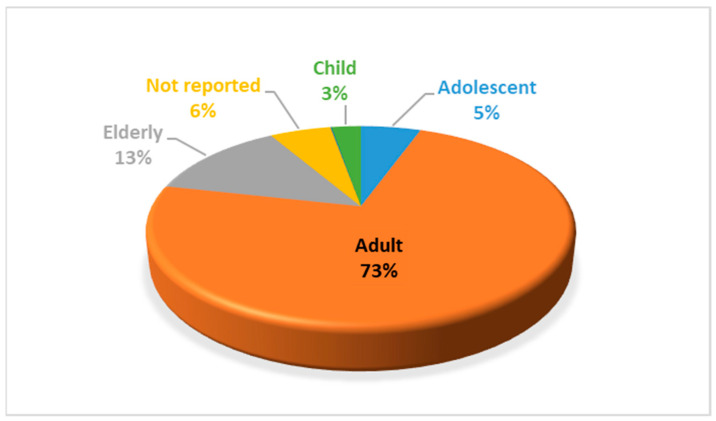
Percentage of self-directed reports by age group.

The mean age was 45.52 years with a standard deviation of 18.89 years, with ages ranging from 1 to 94 years. The highest number of reports was found in the adult group (*n* = 518; 73%) (Figure 4).

**Figure 5:**
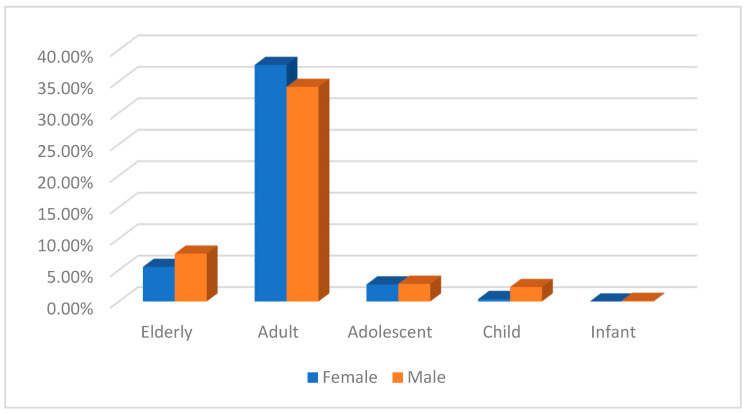
Percentage of self-directed reports by age group and sex.

The distribution of reports according to sex showed that 344 (48.5%) were female and 355 (50%) were male. However, 11 reports did not include gender (1.5%). Figure 5 shows the percentage considering age group and sex, where differences were only observed in the child age group, with 84.20% (*n* = 16) belonging to the male sex compared to 15.8% (*n* = 3) of the female sex.

### 2.3. Self-Directed Adverse Drug Reactions

Of 710 cases, 694 cases were serious (97.7%) and 16 were not serious. These contained 775 reactions of self-directed violence, of which 92 (13%) were fatal, 242 (34.1%) were life-threatening, 216 (30.4%) resulted in hospital admission, 3 (0.4%) resulted in prolonged hospital stay, 8 (1.1%) resulted in patient disability and 281 (39.6%) resulted in a medically significant illness.

The most frequently reported suspected ADRs were for PT suicide attempt (*n* = 319; 41.2%) and suicidal ideation (*n* = 224; 28.9%) (Table 1), HLT suicidal or self-injurious behavior (*n* = 645; 83.2%), HLGT suicidal or self-injurious behavior NCOC (645; 83.2%) and SOC psychiatric disorders (*n* = 649; 83.7%).

In addition, 51% of cases (*n* = 395) recovered and 13.9% (*n* = 108) had a fatal outcome (Table 2).

### 2.4. Drugs Involved

In the 710 notifications, 1053 suspected drugs were recorded; 79 of them were interactions.

The therapeutic groups to which most of the suspected drugs belonged are the pharmacological groups of the nervous system (*n* = 677; 64.5%), anti-infectives for systemic use (*n* = 139; 13.2%), respiratory system (*n* = 57; 5.5%), antineoplastic and immunomodulatory agents (*n* = 54; 5.3%) and alimentary tract and metabolism (*n* = 44; 4.2%) (Table 3).

Within the nervous system, the therapeutic subgroups of psycholeptics (*n* = 231; 21.9%), psychoanaleptics (*n* = 204; 19.5%) and antiepileptics (*n* = 113; 10.7%) were the most reported (Table 4). The active substances of varenicline (*n* = 44; 4.2%), fluoxetine (*n* = 33; 3.1%), lorazepam (*n* = 32; 3%), escitalopram (*n* = 28; 2.7%), venlafaxine (*n* = 23; 2.2%), veralipride (*n* = 22; 2.1%), pregabalin (*n* = 21; 2%) and bupropion (*n* = 19; 1.8%) predominated.

Anti-infectives for systemic use include antivirals (*n* = 116; 11%), especially lamivudine, ribavirin and abacavir, and antibacterials (*n* = 16; 1.5%), mainly fluoroquinolones (*n* = 11; 1.1%) (Table 5).

Likewise, the most reported antineoplastic and immunomodulatory agents were the immunostimulants (*n* = 32; 3.2%) interferon α-2B, peginterferon al α-2A and peginterferon α-2B, and the immunosuppressants (*n* = 15; 1.5%) infliximab, natalizumab, adalimumab, etanercept, apremilast and belimumab.

Within the respiratory system, antihistamines for systemic use (*n* = 10; 0.9%) and agents against obstructive airway diseases (*n* = 40; 3.9%) with montelukast (*n* = 10; 0.9%) and roflumilast (*n* = 21; 2%) were the most frequently reported.

Alimentary tract and metabolism drugs were in fifth place, mostly drugs used in diabetes (*n* = 22; 2.18%). The antiprotozoal agents hydroxychloroquine (*n* = 8; 0.7%) and mefloquine (*n* = 3; 0.3%) were also among the most frequently reported.

Table 6 provides a list of the drugs that were most frequently implicated in self-directed-violence adverse drug reactions. The complete list of self-directed reports for each of the 259 drugs can be found in the Supplementary Materials.

At the top of the list, with 44 reports of adverse drugs reactions, was varenicline, which was implicated in 5 cases of suicide attempt, 34 cases of suicidal ideation, 4 cases of self-harm ideation and 2 of intentional self-harm. It was followed by the psycholeptics lorazepam and veralipride, and psychoanaleptics such as fluoxetine, escitalopram and venlafaxine implicated in several suicide attempts, intentional overdoses, self-injurious behaviors, instances of suicidal ideation and completed suicides, with 14 fatal outcomes overall.

This list includes the phosphodiesterase 4 inhibitor drug (PDE4) roflumilast, reported in 21 cases of self-directed violence (17 occurred in men), including several serious ones: one instance of self-injurious behavior, nineteen of suicidal ideation, two suicide attempts and one intentional overdose.

It also includes the antiepileptic drug pregabalin, with 13 cases in females, associated with one instance of self-harm ideation, eight of suicidal ideation, five suicide attempts, two intentional poisonings, five intentional overdoses and two completed suicides.

The psychoanaleptic, centrally acting sympathomimetic atomoxetine was reported in 12 cases (7 cases in children and 4 in adolescents), 11 of them male, implicated in one instance of intentional self-harm, nine of suicidal ideation and two suicide attempts.

Regarding the qualitative study of the cases, 97.2% had a compatible time sequence, 71.7% of the cases had well-known ADR, 53.2% had drug withdrawal and ADR improvement, 16% had fatal or irreversible ADR, 83.2% had no or unknown re-exposure. In 31.1%, there was no information about the alternative cause, in 30.9% there was enough information to rule it out, and in 29.5% there was an equally or less plausible explanation.

## 3. Discussion

This study was carried out to identify the medicines that may be involved in acts of self-directed violence, as the role of medicines in this type of behavior has been little studied and is often poorly understood. The results of our study indicate that self-direct violence is a phenomenon that is rarely reported, as it represents a very low percentage of all adverse reactions that are reported and corresponds to a relatively small number of medicines. The percentage observed in Spain is similar to studies conducted in other countries [16,26].

Most reactions were observed in the adult group, which is consistent with some studies [26] and shows small variations with respect to others [27]. There were no gender differences except in the child age group, where the majority of reported notifications were from male children.

This study showed a relationship between violence and certain drugs, most of which are related to dopamine, serotonin and gamma aminobutyric acid (GABA), the presence of too much or too little of which could lead to psychiatric conditions such as anxiety and depression. In particular, it seems to be widely accepted that decreased serotonin levels, reduced GABA and increased dopamine are implicated in the etiology of aggression and violence [28].

Among the drugs found to be associated with self-directed violence in this study was the dopaminergic antiparkinsonian drug levodopa. In fact, violent behaviors are part of the dopamine dysregulation syndrome that can complicate the long-term symptomatic treatment of Parkinson’s disease. These include impulse control disorders and behavioral disturbances such as suicidal ideation, suicide attempts and complete suicide. In relation to these medications, a French study found associations with the dopamine agonists bromocriptine, pergolide, piribedil, pramipexole and levodopa [16].

This benzodiazepines lorazepam, diazepam and alprazolam were also found in the reports. These drugs enhance the transmission of GABA, which is a major inhibitory neurotransmitter in humans. However, they also have so-called “paradoxical” effects, such as disinhibition, lack of control and aggressiveness, which are especially likely to occur in susceptible patients [18]. The association between benzodiazepines, hypnotics and sedatives with violence has been found in several studies [16,17,29,30].

The relationship between the selective serotonin reuptake inhibitor antidepressants found in this study (fluoxetine, escitalopram, paroxetine, sertraline) and violence has been extensively explained by Breggin, according to whom these drugs cause a wide range of mental disturbances, such as suicidality, manic psychosis and agitated depression, which can lead to violence towards others and suicide [19]. An association between these antidepressants and violence has been found in several studies [16,17,20,27,31] In addition, some of them also found an association with paroxetine [16,17].

Among the antipsychotic drugs, we found olanzapine, quetiapine, risperidone and paliperidone to have extrapyramidal adverse reactions that have been associated with the development of self-directed violence. Controversy exists regarding these drugs, with some studies linking them to violent behavior and suicide [17,21,26] while others have not [16].

An important finding of this study was the association between self-directed violence and the antiepileptic drugs clonazepam, sodium valproate, levetiracetam, pregabalin and topiramate, corroborating the findings of other research [32]. Regarding topiramate, a study by Nadège Rouve shows that it can cause hyperactivity and aggression in children [16], although no cases of violence associated with topiramate in children were found in this study.

Similarly, an association was found between violence and montelukast, a leukotriene receptor antagonist used for the treatment of bronchial asthma and allergy relief, whose adverse reactions include suicidal ideation, self-injurious behavior, agitation, aggressiveness, anxiety and irritability [17,33,34,35], as well as roflumilast, a phosphodiesterase 4 inhibitor drug (PDE4), which has been linked to suicidal ideation, suicide attempts and completed suicide [22,36]. In this study, most cases of violence associated with montelukast occurred in children under 17 years old, mainly male, whereas all cases related to roflumilast occurred in adults, mainly in males.

Another important finding was the presence of the immunostimulants interferon α 2b, peginterferon α-2a y peginterferon α-2b and immunosuppressant’s infliximab, natalizumab, adalimumab, etanercept and apremilast. Interferon α-2b has the adverse psychiatric reactions of irritability, agitation or paranoia that usually appear after 1–3 months and usually improve within a few days after tapering or withdrawal of the drug [16,17]. Similarly, ribavirin is an antiviral drug used in association with interferon α-2b in the treatment of hepatitis C virus, whose psychiatric adverse reaction profile is similar to that of interferon [16]. Other systemic antiviral drugs implicated in self-directed reports were lamivudine, abacavir, dolutegravir, dolutegravir, efavirenz, tenofovir and emtricytabin. A study from the United Kingdom also reported cases of suicide with efavirenz [26].

Special mention should be made of varenicline and bupropion, widely used smoking-cessation drugs. Varenicline increases the availability of dopamine through the partial antagonism of nicotinic acetylcholine receptors and has been repeatedly associated with aggression, homicidal ideation, and non-fatal suicidal behavior [16,17,26,37]. Bupropion was a selective inhibitor of the neuronal reuptake of catecholamines (noradrenaline and dopamine) which, as in this study, was associated with violence in several studies [26,38,39,40,41].

An association between self-directed violence and the psychostimulants methylphenidate and atomoxetine, which are used for the treatment of attention deficit hyperactivity disorder (ADHD) in children aged six years and older and adolescents, was also found [27,41]. In fact, in this study, the majority of cases were found in the adolescent and child groups.

The use of retinoid for the systemic treatment of acne isotretinoin is also of note, for which a relationship was found and whose technical data sheets list suicidal ideation, suicide attempts and completed suicide as adverse drug reactions, as found in several studies [26,42,43].

The antiprotozoal drugs mefloquine, hydroxychloroquine and chloroquine, which have been used in the treatment of COVID-19, have been associated with suicide attempts and suicidal ideation [26,44].

Finally, several studies have found associations with statins [23,24], but no case was found in this study.

Although these adverse reactions were described for practically all these drugs in their Summary of Product Characteristics (SmPC), it is necessary to emphasize them to improve their prescription, with the aim of preventing such reactions.

This study has many limitations common to studies based on spontaneous reports of adverse drug reactions. One of them is due to underreporting in the spontaneous reporting pharmacovigilance system, and especially in the case of self-directed violence, as it is not easy for most patients to report the belief of an association between a drug and a violent act, especially those with a chronic psychiatric illness or a history of violence, resulting in an underestimation of the magnitude of the problem. Furthermore, the reporting rate may vary depending on the type and severity of the reaction, the drugs involved and whether a new drug is being used. In FEDRA^®^ reports, each report is described using a series of standardized medical terms, and the section where the finding can be explained narratively is generally not filled in, so the quality and detail of the reports varies. It is also necessary to mention the lack of sufficient data in most of the reports on family and/or individual history of violence, alcohol and drug use and dosage of medication used. It is important to increase participation and involve more healthcare professionals in the reporting of adverse reactions. Furthermore, the improvements in reporting are greater when the intervention uses combined strategies [45]. Thus, the results of this study should be interpreted with caution and, knowing the limitations described above, biases such as bias due to the underlying disease of the patients may occur. Additional analysis is considered necessary to consider the different confusion factors. Therefore, although this analysis allows for the detection of potential associations, it requires further evaluation [46]. However, despite all the limitations, as well as the quantitative signal detection methods, these methods are increasingly used for signal generation in pharmacovigilance and drug safety research, as they greatly facilitate the identification of new safety issues or the potential harmful effects of a product [47,48,49,50,51,52,53,54,55]. Thus, these methods contribute to the protection of public health by detecting rare adverse drug reactions, while covering all medicines available on the market.

## 4. Materials and Methods

We conducted a descriptive, longitudinal and retrospective study of spontaneous reports of adverse drug reactions (ADRs) corresponding to self-directed violence included in the database of the Spanish Human Pharmacovigilance System (FEDRA^®^). The study included all reports from 1984 to 31 March 2021. Physical, psychological, verbal and thought acts are defined as self-directed violence.

### 4.1. Selection of Cases

The first step was completed by determining and searching for the set of Medical Dictionary for Regulatory Affairs (MedDRA 24.0^®^) preferred terms (PT) that best define the reaction of self-directed violence, from which spontaneous reports including these terms were searched and selected in FEDRA^®^.

For this purpose, the Standardized MedDRA Queries (SMQ) Narrow Suicide self-harm w usased, which includes the PTs intentional self-harm, self-injurious behavior, suicidal behavior, suicidal depression, Columbia Suicide Risk Assessment Scale (Abnormal), self-harm ideation, self-injurious ideation, suicide attempt, intentional intoxication, intentional overdose and completed suicide. In addition, the SMQ Depression, Suicide and Self-harm included the sensitive PTs feeling of despair, feeling of guilt, negative ideation, emotional distress and decreased self-esteem.

All reports containing one or more MedDRA term (PT) were searched for, identified and evaluated. Thereafter, ADRs were revised. When the reporting form was not sufficiently informative or when the characteristics of ADRs could not be completely defined, the report was not included in the analysis.

### 4.2. Statistical Analysis

The ADRs of self-directed violence were analyzed by applying descriptive statistical techniques for each variable, using SPSS Statistics for Windows, Version 19. (IBM Corp., Armonk, NY, USA).

Among all the variables that were available in the reports, the studied variables were as follows:

- General data on reports: date and seriousness of the report.
- Primary sources: physicians, pharmacists, nurses, other health professionals and consumers, as well as whether patients were in-hospital or out-of-hospital.
- Patient data: gender, age, age group. The age groups were infant (0–1.9 years old), child (2–11 years), adolescent (12–17 years), adult (8–65 years) and elderly (over 65 years).
- ADRs: reactions of self-directed violence (several ADRs can be reported in one report), which were analyzed according to PT, High-Level Term (HLT), High-Level Group Term (HLGT) and System Organ Class (SOC), as well as severity and outcome of ADRs.
- Drugs: drug name, suspected or concomitant drug. Suspected drugs were analyzed according to the Anatomical Chemical Therapeutic Classification System and a qualitative analysis was carried out, including time sequence, prior knowledge, re-exposure and alternative causes.

## 5. Conclusions

This study shows that self-inflicted violence is a real but rare adverse drug reaction related to the use of a small group of medicines. The majority of reported suspected ADRs of self-directed violence were classified as serious, with the majority of these occurring in the 18–65 age group, and with no gender differences except in the child age group. The main drugs with reported adverse reactions of self-directed violence were varenicline, fluoxetine, lorazepam, escitalopram, venlafaxine, veralipride, pregabalin, roflumilast and bupropion. Although there were no unexpected results, this study may serve as scientific evidence supporting the relationship between violence and medications because, although information on self-directed violence exists in the pharmaceutical industry, drug labeling, the medical literature, and publications related to criminal justice, law, psychology, and substance abuse, epidemiological and clinical data are few. It could also be an awareness-raising tool for health professionals, as they should be aware of this association in order to avoid or minimize its consequences. In addition, studies should focus on children and adolescents, as these are particularly sensitive population groups to whom medicines are prescribed that are clearly related to violent behavior. Potential research directions to address gaps in the knowledge about this relationship include studying the psychostimulants methylphenidate and atomoxetine, psychoanaleptics, psycholeptics and montelukast. Prospective longitudinal studies are needed to identify risk, considering more factors (concomitant medication, diseases, etc.). Finally, it is considered necessary to develop new research, using combined strategies such as similar scales to the Health of the Nation Outcome Scales (HoNOS) in participants in clinical trials, that integrates the use of medication into the various factors that influence self-inflicted violence: social, biological, economic, political, cultural and educational factors.

## Figures and Tables

**Table 1:** Distribution of self-directed violence reports in FEDRA^®^ according to Preferred Term of the MedDRA classification.

Preferred Terms of the MedDRA Classification	N	Percentage
Suicide attempt	319	41.2
Suicidal ideation	224	28.9
Intentional overdose	106	13.7
Completed suicide	54	7
Self-harm ideation	29	3.7
Intentional self-harm	18	2.3
Intentional poisoning	10	1.3
Self-injurious behavior	7	0.9
Suicidal behavior	4	0.5
Suicidal depression	4	0.5
Total	775	100

**Table 2:** Outcome of self-directed adverse drugs reactions.

Outcome	N	Percentage
Not reported	141	18.2
Recovering	60	7.7
Fatal	108	13.9
Not recovered	60	7.7
Recovered	395	51
Recovered with sequelae	11	1.5
Total	775	100

**Table 3:** Distribution of self-directed violence reports in FEDRA^®^ according to Anatomical Therapeutic Classification (ATC).

ATC First Level/Therapeutic Group		N	Percentage
A	Alimentary tract and metabolism	44	4.2
B	Blood and blood forming organs	5	0.5
C	Cardiovascular system	21	1.9
D	Dermatologicals	10	1
G	Genito urinary system and sex hormones	2	0.2
H	Systemic hormonal preparations, excluding sex hormones and insulins	5	0.5
J	Antiinfective for systemic use	139	13.2
L	Antineoplastic and immunomodulating agents	54	5.3
M	Musculo-skeletal system	26	2.5
N	Nervous system	677	64.5
P	Antiparasitic products, insecticides and repellents	12	1.1
R	Respiratory system	57	5.5
S	Sensory organs	1	0.1
Total		1053	100

**Table 4:** Distribution of self-directed violence reports of anti-infective for Nervous System drugs.

Nervous System		N	Percentage
N01	Anaesthetics	4	0.4
N02	Analgesics	32	3.1
N03	Antiepileptics	113	10.7
N04	Antiparkinsonians	17	1.6
N05	Psycholeptics	231	21.9
N06	Psychoanaleptics	204	19.5
N07	Other nervous system drugs	76	7.3
Total		677	64.5

**Table 5:** Distribution of self-directed violence reports of anti-infective for systemic use drugs.

Antiinfective for Systemic Use		N	Percentage
J01	Antibacterials for systemic use	16	1.5
J02	Antimycotics for systemic use	1	0.1
J04	Antimycobacterials	2	0.2
J05	Antivirals for systemic use	116	11
J07	Vaccines	4	0.4
Total		139	13.2

**Table 6:** Prescribe individual drugs most often involved in case reports of self-directed violence in the Spanish PharmacoVigilance Database (*n* >10).

DRUG	N	%
Vareniclin	44	4.2
Fluoxetine	33	3.1
Lorazepam	32	3
Escitalopram	28	2.7
Venlafaxin	23	2.2
Veralipride	22	2.1
Pregabalin	21	2
Roflumilast	21	2
Bupropion	19	1.8
Aripiprazol	18	1.7
Paracetamol	16	1.5
Chlorazepate	16	1.5
Olanzapine	16	1.5
Lamivudine	15	1.4
Ribavirin	15	1.4
Alprazolam	15	1.4
Diazepam	15	1.4
Quetiapine	15	1.4
Abacavir	14	1.3
Risperidone	14	1.3
Dolutegravir	13	1.2
Topiramate	13	1.2
Amitriptilin	13	1.2
Levetiracetam	12	1.1
Atomoxetine	12	1.1
Efavirenz	11	1
Clonazepam	11	1
Duloxetine	11	1
Mirtazapine	11	1
Emtricytabin	10	0.9
Tenofovir	10	0.9
Sertraline	10	0.9
Montelukast	10	0.9

## Data Availability

Data is contained within the article and Supplementary Materials.

## Supplementary Materials

The following supporting information can be downloaded at: https://www.mdpi.com/article/10.3390/ph16050772/s1, Excel S1: The complete list of self-directed reports for each of the 259 drugs.
